# A call to action on women’s health: putting corporate CSR standards for workplace health on the global health agenda

**DOI:** 10.1186/s12992-016-0206-4

**Published:** 2016-11-04

**Authors:** David Wofford, Shawn MacDonald, Carolyn Rodehau

**Affiliations:** 1 Meridian Group International, Inc, 2101 L Street, NW, Suite 400, Washington, DC, 20037 USA; 2 Verité, Amherst, MA USA; 3 Meridian Group International, Inc, Washington, DC, USA

**Keywords:** Global health, Family planning, Sustainable development goals, Public-private partnerships, Corporate social responsibility, Policy advocacy

## Abstract

Business operates within a Corporate Social Responsibility (CSR) system that the global health community should harness to advance women’s health and related sustainable development goals for workers and communities in low- and middle-income countries. Corporations and their vast networks of supplier companies, particularly in manufacturing and agribusiness, employ millions of workers, increasingly comprised of young women, who lack access to health information, products and services. However, occupational safety and health practices focus primarily on safety issues and fail to address the health needs, including reproductive health, of women workers. CSR policy has focused on shaping corporate policies and practices related to the environment, labor, and human rights, but has also ignored the health needs of women workers. The authors present a new way for global health to understand CSR – as a set of regulatory processes governed by civil society, international institutions, business, and government that set, monitor, and enforce emerging standards related to the role of business in society. They call this the CSR system. They argue that the global health community needs to think differently about the role of corporations in public health, which has been as "partners," and that the global health practitioners should play the same advocacy role in the CSR system for corporate health policies as it does for government and international health policies.

## Background

This article calls on the global health community to advance global health and development goals for reproductive health and gender equality by taking action on corporate policies and standards related to worker and workplace health as a part of a comprehensive strategy for private sector engagement. Such action requires a different way of thinking about the relationship between business and public health in low and middle income countries and globally. It also requires a different way of thinking about corporate engagement and Corporate Social Responsibility (CSR). The dominant view of the corporate role in health is as external “partners” that provide funding, resources, and know-how to public health and development efforts. Ideally, the outcome of such initiatives benefits both business and society [1], the “win-win” construct that is a staple of CSR and public-private partnerships (PPPs) [2]. This view is reflected in Goal 17 (Partnership for the Goals) of Sustainable Development Goals (SDGs), which aims “to mobilize, redirect and unlock the transformative power of trillions of dollars of private resources” [3]. We propose that global health practitioners not just pursue these valuable public-private partnerships. They should also engage corporations in the public-private policy arena broadly covered by the term CSR and recognize that we are in an era of “new governance” in which corporations, civil society, international bodies, and governments are playing new, intertwined standard-setting, oversight and enforcement roles [4]. This arena of CSR standards is as relevant as PPPs to advancing women’s health policies and goals at the global, national and local levels. Advocacy and engagement on corporate policies and CSR standards should be a core part of the global health’s efforts to achieve SDG Goals 3 (healthy lives and well-being for all) and 5 (gender equality and empowerment) [5]. Such a public-private policy approach situates corporations in their systemic collective relationships and engages new governance levers and a wider range of business incentives that are often missing from the partnership approach.

This public-private policy approach focuses on a specific, but large slice of businesses – multinational and national companies (suppliers and subsidiaries) – that operate in the global economy and under transnational and national rules and standards related to exports. They export everything from cut flowers, cocoa, and coffee to clothes, computers and other consumer goods produced in factory and agribusiness “workplaces” in the developing world. These business are mostly not health care companies, which produce medicines or medical supplies or provide or health services such as private hospitals and health facilities.

So what do these non-health businesses linked to the global economy have do with public health in developing countries? They employ doctors, nurses and other health care staff; they train workers on and implement occupational health and safety practices; they send workers to hospitals and health clinics; they pay into government social security and health insurance schemes programs; and they have a structural and vested interest in the health of their workers and the health services in their communities [6]. They also employ millions of women and men workers who have often migrated from rural areas to find work in the formal economy [7].

The emergence of a truly global economy in the last 30 years has transformed the nature of work and community life in all parts of the world, but most of all in developing countries. Globalization has amplified the role of business in society as well as drawn more women into the workforce. One fundamental change has been the gender make-up of these workplaces. In many industries, women now comprise sizable portions, if not the majority, of the workforce [8]. Yet workplace health policies and practices have not kept up with the changes [9]. Worker and workplace health policies and practices remain defined by the narrow, traditional lens of occupational safety and health (OSH) compliance that was developed decades ago in a different world with men in mind [10]. There has been increasing acknowledgment in regulation of women’s health and safety needs at work, such as protections against pregnancy tests or use of certain equipment for pregnant workers, requirements for day care centers and breast feeding rooms, and restrictions of exposure to chemicals that harm reproductive health [11]. Yet, these are add-ons that do little to address the more fundamental questions of how workplaces should be gender equitable and ensure women’s access to general and reproductive health services and products, what standards should apply to workplace health facilities and providers, how workplace policies should address the specific health needs and workplace hazards needs that represent a particular risk for women, or how occupational health and public health systems should connect. Addressing these questions will also benefit men workers.

Globalization, among other forces, has also helped spur the rise of CSR standards and new governance mechanisms that have changed the structure of business regulation and governance around the world [12]. These CSR standards focused largely on supplementing the weak or weakened governmental regulation of business and workplaces with a private, non-governmental system of standards and accountability mechanisms to address social, environmental and other concerns that shape corporate behavior and workplace practices at the global and local levels [13]. While these standards and mechanisms are often dismissed as purely “voluntary,” this is misleading [14]. In fact, they have evolved – over time and in response to competing strands of social activism on business behavior – into a more structured and overlapping regulatory framework of monitoring and quasi-mandatory or “soft law” compliance for social, environmental and other business issues [15]. This CSR framework is a decentralized network of governance systems that has been put in place and monitored by civil society groups, global organizations and institutions, corporations and national governments [15]. The role of these entities, which independently and in coordination, set, influence, and enforce corporate CSR policies and corporate practices has variously described as an “ensemble regulatory structure” [16] or “polycentric” [17] or “mutual” governance [15]. We would characterize the framework collectively as a global system of CSR standards and governance. For simplicity, we will call it the CSR system.

The global health community has not engaged in the CSR System as an important policy arena relevant to health and the Sustainable Development Goals. The CSR standards of this system address corporate health policy and worker and workplace health, but they are grounded in a narrower OSH approach that is out of step with the changing face of the workforce. The CSR system opens up new ways to engage the private sector in advancing women’s health and related SDG goals in developing countries and promote better health policies for all workers and the workplace.

We are calling for global health engagement in this system, which is highly accessible to policy advocates, but its processes will be new to many practitioners. Section I – the CSR system and its policy processes -- describes the key actors and their interconnected policy roles in the CSR System. Section II – CSR policies, women’s health, and the absent global health advocates -- discusses the lack of global health advocates in CSR policy development and the importance of CSR workplace policies to women’s health. Section III – lessons learned from human rights, labor and environmental advocates -- offers a roadmap for action based on the experience of labor, human rights, and environmental groups in changing policies and hybrid regulatory approaches. Section IV – recommendations for global health advocacy on corporate workplace policies -- proposes five actions for health advocates to change CSR policies and advance women’s health and workplace health practices. Section V – funding, enforcement and business incentives -- addresses questions of cost of policy advocacy and adoption and compliance. Ultimately, the global health community must play the same kind of role for global and national CSR policy that it does for global and national health policy.

## Section I – the CSR system and its policy processes

Evidence of the CSR System is all around us, but few are aware of its relevance to health. People encounter it, for instance, when they buy “fair trade” or “Rainforest Alliance” certified coffee or cocoa at Starbucks. Certification is the public face of this decentralized, networked system of standards, practices, regulatory mechanisms, and compliance and reporting structures that shape corporate policies at the global level and operations at the local supplier level [18]. The system emerged during the last 30 years in response to public concern over the social and environmental effects of globalization, the transnational power of corporations, and the weakening of governmental oversight of business operations in a global context [19]. It is important to understand that Nike does not manufacture its own shoes, nor Gap its clothes, nor Apple its computers. Each company works through a supply chain of usually independent companies, manufacturers and producers (“suppliers”) in developing countries, as part of the new global commercial structure that separates design, marketing and distribution from production and manufacturing [20].

The system developed in response to the various, often contentious, streams of corporate oversight, anti-corruption, human and labor rights, fair trade, environmental and CSR activism. It has no central institution or authority. A range of global – and national – institutions is deeply involved in creating, evolving, and sustaining increasingly sophisticated mechanisms for holding companies (and governments) accountable and establishing policies and programs to protect workers, communities, and the environment. Non-governmental organizations, corporations and their business groups, academics, think tanks, consultancies, legal firms, as well as international and governmental agencies all help set, influence and enforce policies and standards for corporate behavior [21]. Many would not define themselves by CSR, but nevertheless engage in the governance of business’ role in society. The more prominent examples include the United Nations Global Compact, ISEAL Alliance, Social Accountability International, the International Finance Corporation (IFC)/World Bank, Fair Trade Labelling Organizations International, Global Reporting Initiative, Business for Social Responsibility, Worldwide Responsible Accredited Production and Forest Stewardship Council. In general, these actors play one or more of four policy and governance roles in the system at the global and regional levels:

- Standard-setting (eg. IFC, UN Global Compact, Rainforest Alliance, Forest Stewardship Council)
- Policy advice, development, and promotion (eg. BSR, CSR Europe, CSR Asia, Business in the Community, World Business Council for Sustainable Development)
- Enforcement and certification (eg. Rainforest Alliance, WRAP, Fair Labor Association, ILO Better Work Program)
- Reporting and transparency (Global Report Initiative, UN Global Compact, Extractive Industry Transparency Initiative,)

In aggregate, the entities active in the system promulgate a wide range of standards and operating protocols, sometimes in the form of codes of conduct adopted by global corporations as requirements for their suppliers, which are written into procurement contracts or in external certification programs and monitored by brands and external independent organizations.

This system displays several characteristics that drive the process of policy change:

1. *It is competitive, relational and non-linear*. When a standard is improved, other actors are under competitive, institutional, or systemic pressure to improve theirs in order to maintain credibility. When the IFC updates it Social and Environmental Performance Standards for corporate clients, so do more than 80 independent financial institutions and national development finance agencies to keep in alignment [22].
2. *It promotes regular and transparent revisions of policies or “codes*.” The various standards and numerous certification, enforcement, and reporting regimes are updated typically every three-to-five years. The system creates pressure for a timetable and deliberative process of regular policy updates, public input, and transparency for existing standards. Organizations do this for credibility’s sake and for compliance with requirements for regular updates and stakeholder input by the ISEAL Alliance, a membership group to which most standards-setting organizations belong [23].
3. *It promotes integration of policies and standards* so that the standards targeting a specific issue incorporate others. Thus certification regimes developed to ensure fair pay such as fair trade coffee, for example, will address health, transportation, harassment, and housing [24].
4. *It is transnational.* If a global company incorporates a voluntary standard into its supply chain, its suppliers in all countries will be asked to meet that standard. Likewise, certification regimes are transnational. The Rainforest Alliance certification [25], which signifies that a company’s practices in agriculture and other sectors are environmentally sustainable, is the same regardless of country – and any entity seeking certification must meet those requirements (with bi-annual auditing).

With changes in policies and standards also comes the pressure on companies to validate their performance. This has led to the rise of a largely private industry of monitoring, compliance and reporting. Most brands will have an internal compliance staff overseeing suppliers’ fulfillment of business, social and environmental contractual requirements. Many brands hire external auditing firms to undertake site visits to investigate whether suppliers are meeting core labor standards and other requirements [26]. For environmental, labor and other social standards, the supplier may be formally certified or accredited by such independent groups as Utz Certified, Social Accountability International, Worldwide Responsible Accredited Production (WRAP), or the Fair Labor Association. These organizations have their own arrangements for external verification of corporate performance against their requirements before awarding certification or other forms of validation. Such certification regimes verify desired behaviors and leverage market forces to further incentivize good corporate practices and, in theory, punish bad actors. Financial markets are also developing metrics on corporate social and environmental performance [27].

It would be a mistake to think CSR policy development and enforcement operate only at the global level. The “regulatory relationships” under this system “involves dynamic interactions among a variety of actors” both at and between the global and national levels [15]. A few examples must suffice. Corporations play a regulatory role when they agree to external standards, such as the UN Global Compact Principles, and apply them to supply chains in national settings through their contracts and codes of conduct. Civil society plays a regulatory role at the local level through certification regimes with a participating corporation such as when the Rainforest Alliance awards certain Starbucks coffee the Green Frog label after it has certified the environmental sustainable practice of local farms that produced the coffee. Corporations and civil society can play a regulatory role in support of national government policy such as when environmental NGOs and corporate retailers forced soybean traders to sign an agreement not to buy beans from any producer that grew the crop on lands deforested after 2006, thereby using industry corporate CSR commitments, legal agreements and market forces to provide backbone to a new Forest Code enacted by the Brazilian government [28]. Finally, national governments can influence CSR policies and regulation in at least four ways: endorsing them (through CSR information and national guidelines such as in Bangladesh); facilitating them (through subsidies and tax incentives such as the United Kingdom and Denmark giving special market access to Fairtrade-certified products from other countries); mandating them [15] (eg. through procurement and tax policies, such as the Indian CSR law requiring companies to invest two percent of their profits on CSR activities [29]; and partnering with private entities [15].

There is much debate about the merits and performance of this system and the dangers and benefits of the business “citizenship” role in such “new governance” arrangements [4]. But, in one form or another, they are here to stay and will have important influence on hybrid public-private approaches to corporate standards and oversight [30].

## Section II – CSR policies, women’s health, and the absent global health advocates

The global health community has had almost no involvement in shaping or promoting corporate health policies within the CSR system. This is not to suggest that practitioners have ignored the rise of the global economy, CSR and workplace health. The Sustainable Development Goal 17 on partnerships reflects the recognition that many social problems are too large and complex or any one sector to solve on its own in our globalized world and the private sector has a role in the solutions. But the primary approach for engaging corporations in a global economy to address health needs has been through public-private partnerships (PPPs) [31]. In health, PPPs are best defined as “any formal collaboration between the public sector at any level … and the non-public sector … in order to jointly regulate, finance, or implement the delivery of health services, products, equipment, research, communications, or education” [32]. Other private sector approaches, usually with PPP elements, include such market-based ideas as franchising, vouchers, insurance, social marketing, financing and bond mechanisms [33].

The Global Health Community has also engaged in CSR, usually through PPPs, and considered it a form of strategic corporate philanthropy, community investment, or resource mobilization. CSR is a broad term whose definition has evolved since it was first articulated in the late 1940s with changing global business and political environments and changing views of the role business in society [19]. Definitions focus on the integration of social and environmental concerns into business operations and the idea of doing well by doing good [34]. A newer approach, Shared Value, which is cast as not-CSR, is similarly defined as a “management strategy focused on companies creating measurable business value by identifying and addressing social problems that intersect with their business” [35]. Others now use the term “strategic CSR” [36]. Most definitions incorporate the ideas of “enlightened self-interest” and “voluntariness” and link social performance with financial interest and the proverbial business case [37].

These notions of CSR remain valuable and relevant. But they emphasize the centrality of the individual company and business motivations without also capturing the equally significant development of new “ensemble regulatory structures” [16] and standard setting in what we have characterized as a CSR system. This is based on a more comprehensive definition of CSR as “processes of mutual governance between business, civil society, national governments and international organizations” in the management of business’ role in society [15]. It is in this CSR policy sphere where global health advocates and policy-makers have been absent.

The need for CSR policy engagement on women’s and workplace health in developing countries is not an abstract concern. Most industrial or agribusiness workplaces have health infrastructures onsite that can support the expansion and improvement of health services, but are often disconnected from the public health system [38]. Supplier factories and farms are often required by law – and certainly by occupational health practice based on the International Labor Organization’s (ILO) Occupational Health and Safety (OHS) convention – to have occupational health services and providers (health workers, nurses, paramedics, doctors) at the workplace [39]. There is some evidence that these workplaces employ a surprisingly large share of country healthcare workforce. In the 2006 World Health Report, the WHO gave a rough estimate of between 17 percent and 37 percent, but noted the data on these workplace health providers are poor because they are classified under industries that hire them [40]. The report noted: “Excluding them from official counts results in a substantial underestimation of the size of the health workforce and its potential to improve health” [40].

These health providers serve millions of women in global supply chains from Asia to Latin America and the Caribbean to Africa. The data on the number of workers in industry and agribusiness are limited as with the data on company health providers. The International Organization for Migration (IOM) estimates that 105 million people, half of which are women, leave their homes to find work in other countries [41]. There are many more who migrate internally to urban centers to work for companies supplying products to the global economy [41]. In Bangladesh, the garment sector employs about four million workers, mostly women [42]. Cambodia has an estimated half a million workers in the garment industry, which employs about 25 percent of women in the country between the ages of 19 and 29 [43]. African countries are also connected to the global standards system typically through extractive industries (e.g. mining and oil) as well as agribusiness (e.g. cut flowers, tea, coffee, cocoa and palm oil) [44].

Thus, across the world, millions of women have left their rural homes and social structure in search of employment usually in urban centers and across borders [45] and work global supply chains of agribusiness and in industry. They are low paid, often living in dormitories or with friends, under pressure to send remittances home, and often disconnected from family and public support systems. These women’s health needs are significantly different from those of men workers – and their general health can be harmed by the conditions at work and restrictive policies and practices that ignore these needs. There has been little attention paid to their need for access to general and reproductive health services and products, the poor quality of workplace infirmaries and practices of workplace health providers, and the poor sanitary conditions at work. CSR policies and business practices do not take women’s health seriously as a workplace health priority, a fundamental business interest, and a governance issue.

What would better CSR policies and practice for women’s and workplace health in developing countries look like? To start, they would not mean that every corporation and supply chain company must run a primary care facility at the workplace and offer family planning. They would mean that corporate policies and workplace practices must protect women’s health, not just safety, enable women’s access to health services, including reproductive health, and ensure the quality of care of providers and facilities on site. In poor countries, women’s health at work is compromised by a range of common operational practices. For instance, restrictions on breaks, restrooms and water can cause urinary tract infection [46]. Lack of menstrual hygiene products and clean, private restroom can cause gynecological infections [47]. These are issues that do not affect men and do not make the OSH list of concerns.

Access to health services, particularly reproductive health services, are critical for women workers, many of whom face what has been called “the double burden of work,” [48]. Even if available services exist where they work or live, the long hours at work often mean women cannot access services after work – and their domestic duties further limit access. Many are young women who have need for family planning education, products and services. Company policies and de facto practices on leave directly impact their access to these health services.

Finally, very little attention is paid to the quality of workplace health facilities and staff that are usually required by law. These facilities and staff are primarily responsible for addressing workplace injuries. There is no policy expectation for these facilities to meet some level of public health standard for hygiene, confidentiality, or patient-centered care [49]. There are equally low expectations for health care providers. The limited qualifications and skills – and expectations – of workplace nurses is common in many factories and farms. A Business for Social Responsibility (BSR) study in 10 factories in Bangladesh found 40 percent of the nurses did not have nursing diplomas. Bangladesh law and corporate code compliance requires factories to have diploma nurses. BSR found “a discernible mismatch between the training received by the nurses and the actual health needs in [garment] factories” [50]. Nurses were under-utilized, unprepared to handle emergencies, and not trained to address the RH needs of the predominantly female workforce [50]. Neither industry nor government has made much effort to build nursing skills or ensure qualifications relevant to the factory workforce.

These are policy issues that global health practitioners should address through the CSR System. But they have not viewed it as relevant to public health goals or system strengthening. The most active stakeholders in corporate accountability activism have come from environmental, human, or labor rights backgrounds [51] – and health is not their primary concern. As a result, the policies promoted by current stakeholders within the system do not address workplace health standards and practices or consider the significant health needs of women and men workers. They look to occupational safety and health conventions [9] to guide their thinking about health. These stakeholders view occupational health as a settled issue concerned with reducing the numerous physical dangers faced by workers, such as exposure to toxic chemicals, dangerous machinery, fire hazards and building safety. Most corporate OSH standards and related industry efforts, therefore, focus on safety issues aimed at reducing workplace injuries: adequate ventilation and lighting, fire extinguishers and exits, structural safety, and first aid supplies [49].

When companies comply with OSH standards, this has little effect on quality health services by health staff and worker access to quality health services onsite and offsite. OSH compliance monitoring does not address whether workplace health services meet the AAAQ (available, accessible, acceptable, appropriate and of good quality) rights framework for public health facilities and staff [49]. There is little recognition that AAAQ might be relevant to workplaces. No one enforces basic clinical standards, such as confidentiality and privacy, knowledge and skills of health providers on health issues relevant to the workforce, or practitioner hygiene practices like handwashing or disposal of medical waste. There is very little oversight of both policies and actual practices that enable workers to seek care onsite or offsite and without retribution from managers.

Better health policies and practice at the workplace require the leadership of public health practitioners in CSR standards and governance.

## Section III – lessons learned from human rights, labor and environmental advocates

A common response to this situation is to argue for government policy change and stricter enforcement, and that certainly should be done. Yet, it would be a mistake not to harness the incentive power of CSR policies that are set and enforced under the CSR System or to assume that public policy change alone will be quicker or more effective. Moreover, this misses the point in this era of mutual governance of the inter-relationship of CSR and government governance, which overlap and thereby present new opportunities for addressing regulatory voids and spurring creative approaches to advancing for women’s health.

The experience of environmental, labor and human rights advocates is instructive. The approaches below provide a useful roadmap for global health practitioners for collective action:

- **Produce data and documentation to spur policy change**. Environmentalists have been leaders in using scientific data to document the impact of industry on climate change and advocate for stronger measures by corporations and their supply chains to limit environmental impacts. Most major companies now have programs to limit the use of raw materials and water and emissions of pollutants. Documentation in Nigeria and elsewhere by human rights groups produced evidence of killings by private security forces employed by extractive companies [52]. Advocacy supported by documentation led to these groups and the governments of the United Kingdom and the United Stated developing the Voluntary Principles for Security and Human Rights in the 2000 [53]. As human rights are traditionally a state responsibility, the principles opened the door to including transnational corporations within a framework of human rights responsibility. Five years later, the United Nations Human Rights Council launched a process to define the role of business in human rights. In 2011, the United Nations adopted the voluntary Guiding Principles on Business and Human Rights, which assigned the following responsibilities: the state duty to protect human rights against third party abuses; the corporate responsibility to respect human rights; and greater access by victims to effective remedy, both judicial and non-judicial [54].
- **Target key organizations for wide-scale corporate policy adoption**. The policy actions of many institutions and organizations influence other institutions. The United Nations’ adoption of the Guiding Principles on Business and Human Rights caused many others to incorporate them, including the UN Global Compact, the World Bank, and the Organization for Economic Cooperation and Development (OECD), to be in policy alignment [55]. These policy changes are transnational.
- **Use collective action and creative corporate regulatory arrangements to address problems where public policy change is not possible or government regulation enforcement capacity is weak**. Labor and environmental advocates have developed new hybrid mechanisms for oversight of workplace activities in the many cases where government agencies lack the political will and resources. We noted above the Soy Bean Moratorium in Brazil developed by environmentalists. Another example is in Florida where the Coalition of Immokalee Workers launched the Fair Food Program (FFP) in 2011, a model to improve the wages and lives of tomato workers that includes a set of industry standards, worker training on labor rights, external auditors to certify growers, and a wage premium paid by tomato buyers like Wal-mart and Taco Bell to producers, that is passed on as higher wages to workers. A key enforcement mechanism is the market and brand leverage, which was made possible by a set of corporate codes and policies. But participating buyers sign legally binding agreements not to buy from any growers that do meet the FFP code of conduct [56].
- **Develop tools and instruments the drive CSR policy change and compliance**. Various stakeholders are creating a range of instruments to help business report on their social and environmental activities (Global Reporting Initiative) [57], assess their impacts (the Danish Institute for Human Rights compliance assessment tool) [58] and implement policies (UN Global Compact’s SDG Compass) [59].
- **Seize opportunities to expand policies within standards beyond a single issue**. For instance, the Roundtable on Sustainable Palm Oil (RSPO), which established the primary certification system for environmentally sustainable palm oil, had focused only on improving environmental performance and reducing rainforest destruction until civil society advocacy recently forced the RSPO to adopt entirely new requirements on human rights and labor [60]. Labor groups successfully advocated for inclusion of labor rights to environmental focus of the Responsible Jewelry Council standards [61], similarly convinced the Electronic Industry Citizenship Coalition to extend its standards to cover labor recruitment [62].

By working collectively and through coalitions, advocates for stronger corporate policies and practices on the environment and labor and human rights have leveraged this system (which they also helped create) to further their goals.

## Section IV – recommendations for global health advocacy on corporate workplace policies

The global health community has extensive experience in policy development and advocacy in the public sector. Practitioners draft health policy, generate evidence, create coalitions to advocate for change, organize media campaigns, and advocate for change with national government and international health bodies. The CSR System may be an unfamiliar policy arena, but the process of policy development and change is similar. We recommend that practitioners take the policy know-how and experience in PPPs they already have and advocate for women’s health in CSR policy sphere.

We recommend five areas for action:

1. **Put CSR standards for women’s health on the global health agendas a core issue**.
  This requires public and private donors and leading health organizations to view CSR policy as a as a strategic focus area and make it one of the central topics in existing forums and convenings. Major conferences, such as the International Conference on Family Planning and Women Deliver should consider CSR policy as deserving exposure in plenaries and incorporate it into thematic tracks. This also means participating in the global policy discussions about corporate citizenship and responsibility in influential venues such as the World Economic Forum, BSR and Aspen Institute as thought leaders for women’s health in CSR. The focus for these discussions on CSR policies should be SDG 8 (decent work) as much as SDG 17 (partnerships).

2. **Target key institutions that can drive CSR policy change**
  Global health organizations should target entities whose policy changes have the greatest influence and reach. Examples include global institutions like the International Finance Corporation of the World Bank Group (with social and environmental performance standards) and United Nations (Global Compact and Guiding Principles on Business and Human rights), certification organizations like Rainforest Alliance and Fair Trade International, industry groups like the Electronics Industry Citizenship Coalition. In many cases, advocacy may not be about changing the standard but rather influencing the requirements, indicators, and guidelines for meeting the standard. For instance, corporate implementation of the UN Guiding Principles on Business and Human Rights directs companies to do a risk assessments of their potential complicity in or direct violation of workers’ human rights. The process should include corporate guidance on how to assess the potential harms and possible human rights violations to workers’ reproductive and women’s health rights in their supply chains.

3. **Leverage existing relationships and existing issues**
  Global health works closely with leading environmental groups on a population, health and the environment (PHE) agenda. Environmental groups play advocacy roles within the CSR System, but do not advocate for CSR policies, for instance, that improve worker access to family planning services. Practitioners should leverage these relationships to address jointly corporate workplace policies in global supply chains as a PHE strategic focus. Similar relationships exist for women’s empowerment organizations, which often have the attention of global corporations. Yet, family planning and reproductive health are not yet considered essential elements of corporate initiatives to empower women workers. The global health community should also find common ground with labor advocates around such issues as living wages and the impact of health care costs on women workers’ take home pay.

4. **Generate data, evidence and tools related to workplace health policies and practices**
  One of the biggest deficits of data and documentation is the size of the health workforce in commercial firms in developing countries. Leading public health donors should engage the International Labor Organization and national governments to improve the data collection. Documentation is needed on what workplace policies and practices are effective in increasing access to health services, including the use of transport vouchers, referral mechanisms, and onsite capacity building. More rigorous research is needed on the benefits for both business success and worker health as well as for surrounding communities from better health practices. Finally, tools are needed to help stakeholders improve workplace health practices and produce better metrics on worker and women’s health that can be incorporated in CSR reporting.

5. **Promote CSR policy linkages in the workplace between occupational and public health**
  New arrangements need to be developed to bring industrial and agricultural workplaces in line with public health standards and practices. This poses a structural challenge at the national level as ministries of labor oversee health in the commercial workplaces and ministries of health oversee health in all other public and private facilities. Thus, occupational health and public health occupy different policy spheres that are poorly integrated – to the detriment of both. The WHO’s Global Plan of Action on Workers’ Health (2008-2017), Adelaide Statement on Health in All Policies (2010), and Health in All Policies Framework for Country Action (2013) are important signposts for a new direction [63]. Creative CSR policy and governance approaches can support improved women’s heath practices at the workplace and build new linkages between workplace health facilities and public health resources. In Bangladesh, an example of such linkages is the Bangladesh Garment Manufacturers and Exporters Association’s agreement with the Directorate General of Family Planning to enable qualified clinics in BGMEA-member factories to receive family planning products [64]. In every country, the response to addressing workplace health standards and building linkages health will be different.

## Section V – funding, enforcement and business incentives

No doubt these recommendations, particularly research and certain advocacy activities, require new funding from donors and the private sector. Yet it is important to recognize that engagement in the CSR System and policy changes will also use or repurpose resources that already exist, some of which already address private sector engagement. Adding CSR policy to the forums and conventions is not an added cost; it is an added dimension. The costs of advocacy with global institutions is relatively low and in some case may be integrated into other interactions and activities with these organizations. Furthermore, much of the development of policy proposals can happen remotely and online.

As for the funding of improved workplace health practices, it is important to note that many of the resources and funding already exist onsite. Manufacturers and agribusinesses already employ health providers, human resources staff, compliance officers, training activities and related business structures. The question is how to use these existing resources in more effective ways that expand women workers’ access to quality health services onsite or off. This effort will also entail new costs, which will need to be borne by corporations, suppliers and public entities in different proportions varying by country and industry.

A full description of enforcement of new health policies is beyond the scope of this paper, but some of the key mechanisms have been highlighted in section two. The CSR system requires a new way of thinking about enforcement as it leverages the interaction of many different layers of oversight, from traditional government oversight (eg. workplace inspectors) to non-governmental oversight: corporations (compliance officers), NGOs (eg. certification regimes, reporting mechanisms), workplace personnel (eg. compliance, human resources and health staff). This also leaves out the most important emerging non-governmental enforcement– market and financial mechanisms that influence corporate behavior base through lending protocols and rating systems. Again, these enforcement structures already exist. The question for global health advocates is how these different layers of enforcement can reinforce each other to advance women health in the workplace and in society.

It would be natural to think the CSR advocacy leads to conflict with the corporate partners or becomes associated with anti-corporate activism. Any effort to change policy creates tensions among interested stakeholders. Yet, in the case of advocating for women’s health and better workplace health standards in CSR policies, such changes align with many corporates existing commitments and are in business’ self-interest. The private sector played a major role in the development of the SDGs, and more and more global and national corporations have committed to supporting their implementation. CSR advocacy on women’s health responds directly to SDG goals 3 (healthy lives), 5 (gender equality), 8 (decent work) and 17 (partnerships), and others. This is also true for corporate commitments on many other standards.

Health advocacy also connects to the CSR notion of enlightened self-interest and business performance. A range of workplace health programs have shown that it is in the self-interest of business to address worker health. Companies that invest in worker health, not just in the reduction of workplace injuries, are more likely to see a return on investment (ROI) in the form of increased productivity, improvements in recruitment and retention, and decreases in turnover rates [65]. Globally, research into workplace health programs have also documented improvements in staff morale and employee-manager communication, worker engagement as well as declines in short-term disability and worker compensation and employee “presenteeism” (ie. workers onsite but not productively engaged) [66]. Thus, a growing body of evidence is showing that healthy workers are good for a company’s bottom line [67].

## Conclusion

This article has called on the global health community to take action in a new policy arena that shapes corporate practices on women’s and workplace health as a core strategy of achieving the Sustainable Development Goals. The global health community engages corporations – and CSR – through public-private *partnerships*. We have argued this value approach leaves out the importance of CSR policies as part of a “new governance” framework that shapes corporate *policies* and business’ evolving role in society. We have called this framework the CSR system, which is based on a definition of CSR as a set of “processes of mutual governance between business, civil society, national governments and international organizations” in the management of business’ role in society. This encompasses the more common definitions of CSR based on business motivation. In this system, civil society, businesses, global institutions and governments set, monitory, and enforce standards and practices on social, environmental and other issues. But health has not been a major focus because the system leaders have been environmental, human rights, and labor advocates, not health practitioners. We argue that the global health community needs to engage in CSR policies relating to SDG goals on women’s health and workplace practices and make five recommendations:

1. Put CSR standards for women’s health on the global health agendas a core issue
2. Target key institutions that can drive CSR policy change
3. Leverage existing relationships and existing issues
4. Generate data, evidence and tools related to workplace health policies and practices
5. Promote CSR policy linkages in the workplace between occupational and public health

The world of corporate governance and regulation is evolving rapidly under this system as business, civil society, global institutions and governments are crafting new approaches and reshaping old ones to tackle public needs [30]. Global health needs to be part of this change. This requires the global health community to take action and advocate for health just as activists for the environment, labor, and human rights have done.

## Abbreviations

BSR: Business for social responsibility
CSR: Corporate social responsibility
FFP: Fair food program
IFC: International Finance Corporation
ILO: International Labor Organization
NGOs: Non-governmental organizations
OECD: Organization for Economic Cooperation and Development
OSH: Occupational safety and health
PHE: Population, health and the environment
PPP: Public-private partnerships
ROI: Return on investment
RSPO: Roundtable on sustainable palm oil
SDGs: Sustainable development goals
UN: United Nations
USAID: United States Agency for International Development
WHO: World Health Organization
